# Massive duodenal variceal hemorrhage in a patient with prior Roux-en-Y gastric bypass

**DOI:** 10.1016/j.radcr.2021.08.002

**Published:** 2021-08-26

**Authors:** Kiran Sinjali, Chris Bent

**Affiliations:** a University of California, Riverside School of Medicine, SOM Education Building, 900 University Ave, Riverside, CA, 92521 USA; b Department of Radiology, Riverside University Health System, 26520 Cactus Ave, Moreno Valley, CA 92555 USA

**Keywords:** Duodenal variceal bleeding, Ectopic varices, Percutaneous transhepatic embolization, Transjugular intrahepatic portosystemic shunt, Roux-en-Y gastric bypass, Portosystemic pressure gradient

## Abstract

Duodenal variceal bleeding is a rare form of variceal bleeding which may be fatal if left untreated. There are no specific guidelines available for their treatment. Medical management, surgical, endoscopic, and interventional radiological procedures have been utilized with varied outcomes. In this case summary we report the successful management of duodenal variceal bleeding in a patient with prior Roux-en-Y gastric bypass . The patient with history of cirrhosis presented with acute gastrointestinal bleeding. Esophagogastroduodenoscopy and colonoscopy could not locate the source of bleeding. Computed tomography of the abdomen demonstrated a large duodenal variceal complex. Interventional radiology (IR) treated the patient with a combination of percutaneous transhepatic embolization and subsequent transjugular intrahepatic portosystemic shunt . No recurrence of gastrointestinal bleeding was noted at follow up. This case demonstrates that percutaneous transhepatic embolization along with transjugular intrahepatic portosystemic shunt may be effective treatment of duodenal variceal bleeding.

## Background/Introduction

Gastrointestinal varices are abnormal, enlarged submucosal veins caused by portal hypertension and most commonly develop in patients with cirrhosis. About 50% of patients with cirrhosis develop varices in gastroesophageal region whereas only 3.5%-8.5% have varices develop elsewhere in the gastrointestinal (GI) tract. These are known as ectopic varices [1,2,3]. Variceal bleeding occurs in 25%-35% of patients with cirrhosis with mortality of up to 30% [4]. Ectopic varices account for only 1%-5% of all variceal bleeding. Duodenal varix bleeding when it occurs has a higher reported mortality of up to 40% [2,5,6]. Due to its rarity, there is a relative lack of evidence for effective treatments for ectopic varices. Current treatment methods include medical management, surgical interventions, endoscopic band ligation, endoscopic sclerotherapy, percutaneous transhepatic embolization, and transjugular intrahepatic portosystemic shunt (TIPS) with varied outcomes [3,7]. We present the imaging findings of a patient with duodenal variceal hemorrhage in a patient with history of Roux-en-Y gastric bypass (RYGB) which was successfully treated with percutaneous transhepatic embolization followed by TIPS.

## Case presentation

A 51-year-old female was admitted to intensive care unit for severe anemia secondary to acute GI bleeding after presenting to the emergency department for melena and epigastric abdominal pain. She reported nausea, abdominal distention, and dark red stools but did not have fever, chest pain, shortness of breath, vomiting, or other relevant symptoms. Medical and surgical history were significant for decompensated alcoholic cirrhosis, RYGB, end stage renal disease on intermittent hemodialysis, and gastrojejunal anastomotic ulcer bleeding 3 months prior to admission for which she underwent outpatient endoscopic clip placement.

**Fig. 1:**
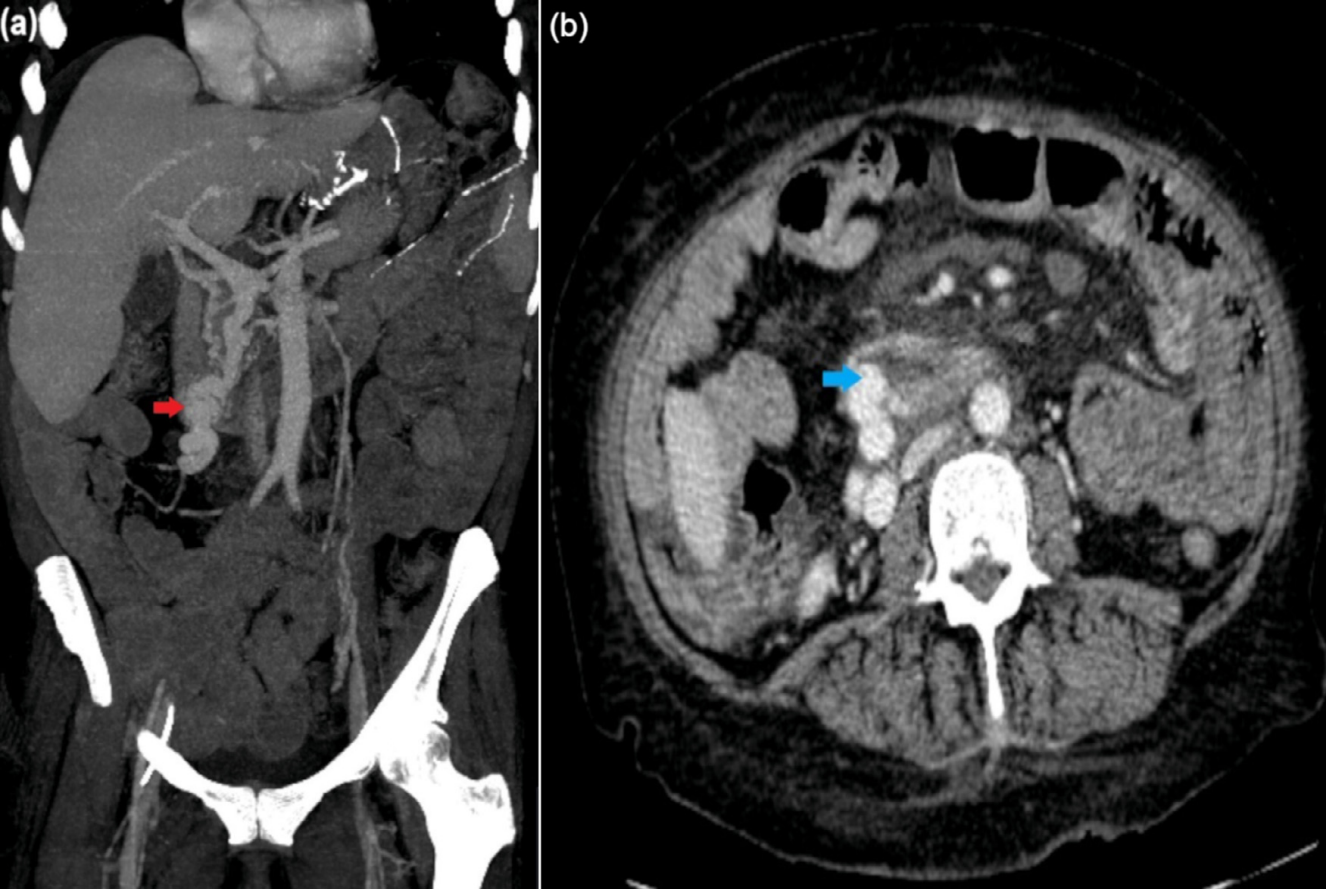
CT images prior to percutaneous transhepatic embolization and TIPS. (A) Coronal post intravenous (IV) contrast CT image through abdomen and pelvis demonstrating a large duodenal varix (red arrow) with periduodenal edema. (B) Axial post IV contrast CT image demonstrating duodenal varix protruding into duodenal lumen (blue arrow).

Upon presentation, the patient was hypotensive, her hemoglobin concentration (Hb) was <2.5 g/dL (normal 11.1-15.9 g/dL), total bilirubin >10 mg/dL (normal 0.0-1.2 mg/dL) and model for end-stage liver disease with serum sodium (MELD-Na) score was 25 with estimated 90-day mortality of 14%-15% [8,9]. Medical management for GI bleeding was initiated. Gastroenterology performed an EGD identifying a gastrojejunal anastomotic ulcer and esophageal varices which were banded. Neither endoscopically demonstrated high-risk stigmata of recent hemorrhage. Patient also underwent colonoscopy which was unremarkable. CT of abdomen and pelvis demonstrated a large duodenal varix (Fig. 1) with periduodenal edema. IR was consulted for management of presumed ectopic duodenal variceal hemorrhage.

**Fig. 2:**
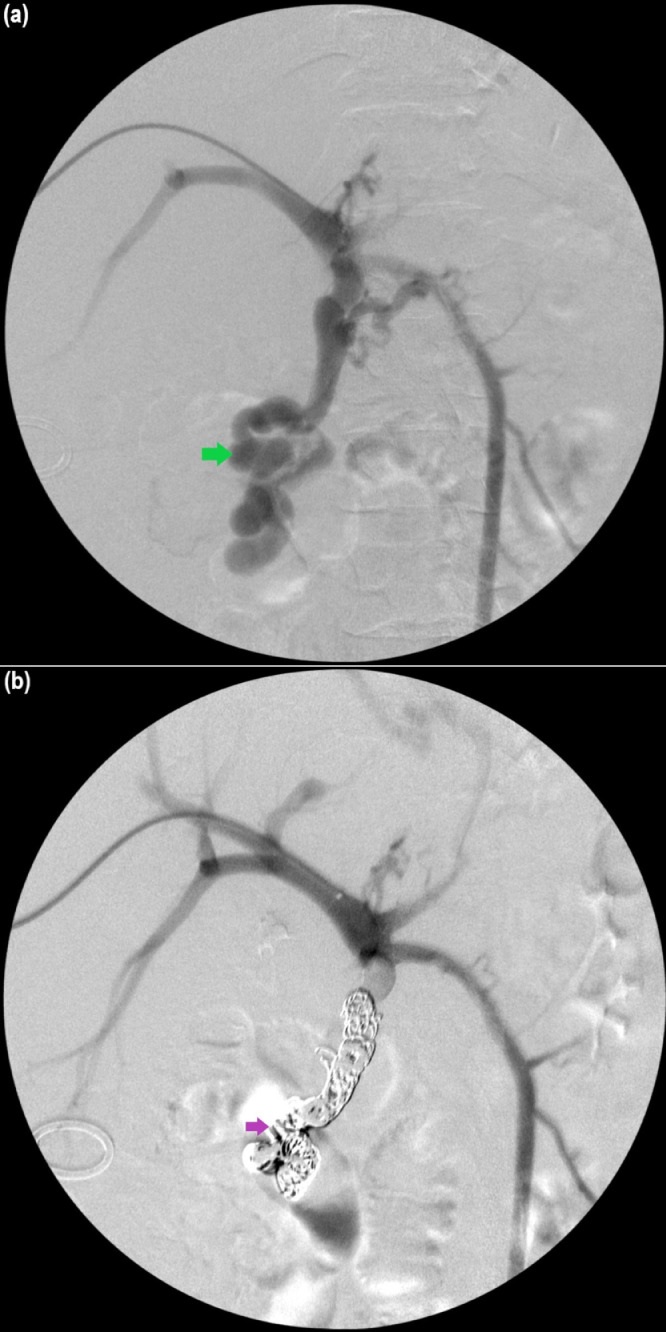
(A) Transhepatic portal venogram demonstrating large duodenal varix (green arrow). (B) Venogram demonstrating successful coil embolization of duodenal varix (pink arrow) after percutaneous transhepatic embolization.

**Fig. 3:**
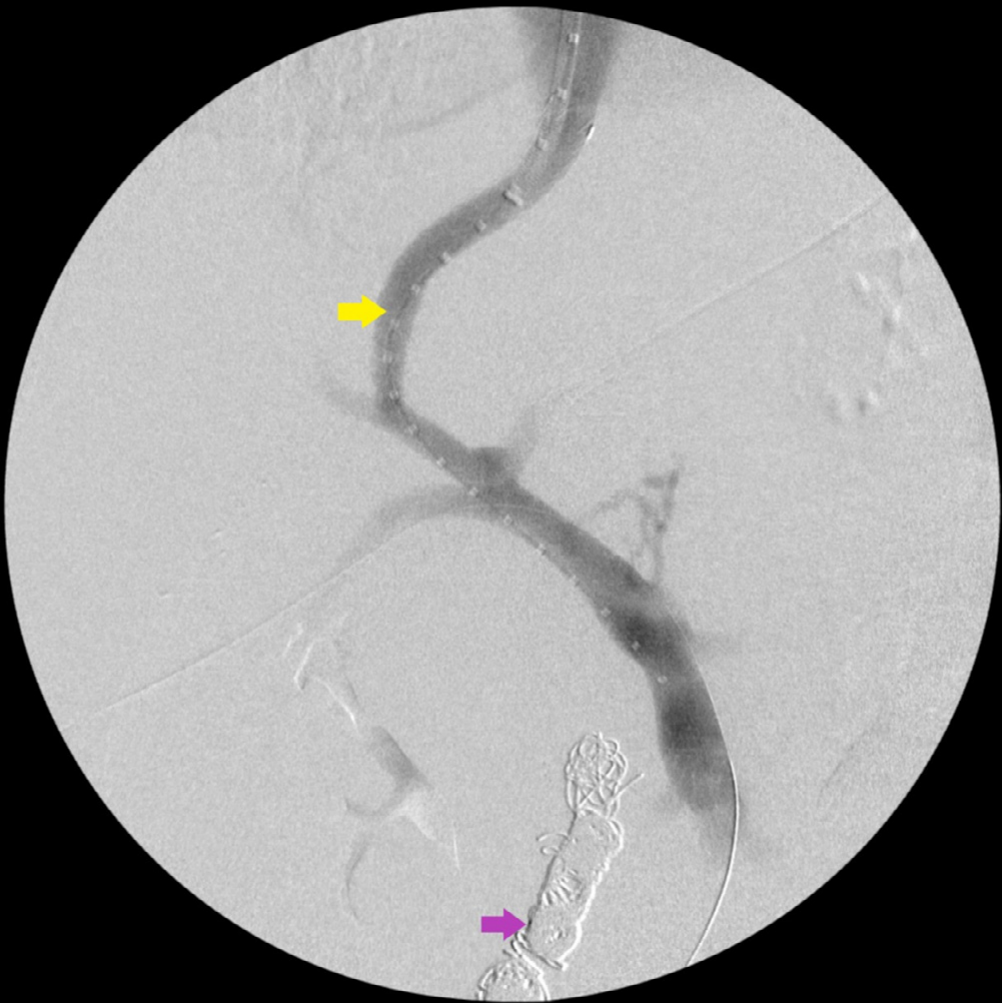
Post TIPS placement portal venogram demonstrating patent TIPS (yellow arrow), and embolized varix (pink arrow).

After multidisciplinary discussion with surgery, gastroenterology, and IR services, TIPS was initially deferred due to elevated MELD-Na score and hyperbilirubinemia and associated risk of fulminant liver failure. Due to prior RYGB endoscopic options were limited as endoscopes could not reach the duodenum. Transhepatic portal venogram with percutaneous transhepatic embolization of the large duodenal varix (Fig. 2) was performed. The patient's total bilirubin decreased to 4 mg/dL (normal 0.01.2 mg/dL) and MELD-Na score decreased to 16 with estimated 90-day mortality of <2% over the next several days, however, intermittent episodes of slow GI bleeding continued to occur requiring transfusion [8,9]. Repeat EGD redemonstrated the treated esophageal varices and non-bleeding gastrojejunal ulcer without source of bleeding. Given improvement in liver function, TIPS was then performed with decrease in portosystemic pressure gradient from 14 mm Hg to 9 mm Hg (normal ≤ 5 mm Hg) (Fig. 3) [10]. The patient's Hb remained stable at approximately 7.4 mg/dL (normal 11.1-15.9 g/dL) with resolution of bleeding and the patient was subsequently discharged [8]. The patient was seen in gastroenterology clinic with interval CT of the abdomen and EGD follow up with no recurrent episodes of GI bleeding or significant varices after 5 months.

## Discussion

Gastrointestinal varices form in response to portal hypertension. Normally, blood drains from abdominal organs into the portal venous system that courses through the liver before returning to the systemic venous system via hepatic veins. There are alternative collateral routes that are non-functional in normal situations due to their high resistance to blood flow compared to the portal venous system. When intrahepatic vascular resistance increases as a result of cirrhosis, portal hypertension can develop leading to the recanalization and/or hypertrophy of the typically unutilized collateral pathways [11]. Portosystemic pressure gradient is estimated by hepatic venous pressure gradient (HVPG) which is a function of wedged hepatic venous pressure (WHVP) and free hepatic venous pressure (FHVP), defined as HVPG = WHVP – FHVP [11]. Significant elevation in the portosystemic pressure gradient to >10 mm Hg and >12 mm Hg (normal ≤ 5 mm Hg) increases risk for variceal development and bleeding, respectively [10]. Studies have shown portosystemic communications via different routes, such as gastroesophageal plexus to azygous-coronary system, pancreatoduodenal venous arcade to inferior vena cava, hemorrhoidal plexus. The most common location of varix formation is gastroesophageal region. Ectopic varices include duodenal, rectal, or peristomal varices, and prior abdominal surgery predisposes patients to develop ectopic varices [12]. As varices increase in size, the chances of rupture increase [4]. There are no specific guidelines for management of ectopic variceal bleeding, including duodenal varices. Surgical management is less frequently used due to high mortality and morbidity [4]. Apart from medical management, endoscopic interventional procedures, such as endoscopic band ligation and endoscopic sclerotherapy have been frequently used individually or in a combination to treat ectopic varices. Neither of them has a clear superiority. Endoscopic band ligation is associated with higher variceal recurrence whereas endoscopic sclerotherapy is associated with higher rebleeding. Metachronous combination of endoscopic band ligation and endoscopic sclerotherapy has potential for better outcome compared to individual treatments [13,14]. In addition, interventional radiological procedures such as TIPS, balloon occluded retrograde transvenous obliteration and percutaneous transhepatic embolization have also been used.

When anatomically viable, balloon occluded retrograde transvenous obliteration has reported success rates of up to 89% in controlling bleeding, lower risks of hepatic encephalopathy but has higher risks of rebleeding compared with TIPS [15]. Similarly, percutaneous transhepatic embolization also has success rates of about 80% in controlling initial bleeding but has higher rates of rebleeding [16]. TIPS has been shown to reduce portosystemic pressure gradient and has also been more effective in preventing recurrence of varices and rebleeding compared to endoscopic interventions. However, TIPS is associated with risks of developing hepatic encephalopathy, or liver failure especially in those with elevated MELD-Na scores. Nevertheless, a 25%-50% and >50% decreases in portosystemic pressure gradient were found to reduce risks of rebleeding to 7% and 1%, respectively [17]. Percutaneous transhepatic embolization combined with TIPS was found to be a superior treatment compared to either TIPS or percutaneous transhepatic embolization alone in terms of rebleeding in retrospective cohort studies [16,18]. Mengying Liu *et al.* reported a case of 54-year-old female with duodenal variceal bleed, primary biliary cirrhosis and Child-Pugh class B who was treated with TIPS and venous embolization together. The patient had no rebleeding or residual duodenal varix at 24 months follow up [3]. In our case, the patient was treated with percutaneous transhepatic embolization followed by TIPS due to initial contraindication, but still had favorable outcomes.

Despite lack of randomized controlled trials and strong evidence for effective treatment of duodenal variceal bleeding, the index case demonstrates that percutaneous transhepatic embolization along with TIPS may be effective in stopping ectopic duodenal variceal hemorrhage. Combination of interventional radiological procedures should be strongly considered by the patient care team as treatment option for duodenal variceal bleeding, especially in those with prior RYGB where endoscopic options are precluded.

## Patient consent statement

The images included in this report are anonymous and do not allow for identification of the patient. The overseeing institutional review board waves the need for informed consent in case reports which do not include identifying information.
